# Kazakh ThromboTest for intraoperative thrombosis risk assessment during stent-assisted cerebral aneurysm treatment: a single-center clinical study

**DOI:** 10.3389/fneur.2025.1687591

**Published:** 2025-11-04

**Authors:** Mynzhylky Berdikhojayev, Aiman Maidan, Shayakhmet Makhanbetkhan, Rajeev Sivasankar, Maxat Mussabekov, Dimash Davletov, Roger Barranco Pons, Marat Sarshayev

**Affiliations:** ^1^National Hospital of the Medical Center of the Presidental Affairs Administration of the Republic of Kazakhstan, Almaty, Kazakhstan; ^2^Department of Interventional Neuroradiology and Neurosurgery, Instituto Médico ENERI, Clínica La Sagrada Familia, Buenos Aires, Argentina; ^3^Department of Imaging & Interventional Radiology, Fortis Hospital, Mumbai, India; ^4^Asfendiyarov Kazakh National Medical University, Almaty, Kazakhstan; ^5^Al Qassimi Hospital, Dubai, United Arab Emirates

**Keywords:** Kazakh ThromboTest, stent assisted coiling, clopidogrel resistance, VerifyNow, intraoperative thrombosis, dual-antiplatelet therapy, endovascular neurosurgery

## Abstract

**Background:**

Stent-assisted endovascular treatment of cerebral aneurysms enhances long-term occlusion rates but carries a risk of thromboembolic complications, often influenced by resistance to antiplatelet therapy. Conventional platelet function tests, such as VerifyNow^™^, have limited predictive value and accessibility, particularly in resource-limited settings. The Kazakh ThromboTest (KTT) was developed as a real-time intraoperative method to detect thrombotic risk and guide antiplatelet management during neurointerventional procedures.

**Methods:**

We retrospectively analyzed 284 patients who underwent stent-assisted aneurysm treatment at a single neurovascular center in Kazakhstan between 2020 and 2023, all of whom received dual antiplatelet therapy. The Kazakh ThromboTest (KTT) was performed intraoperatively in every case, and preoperative platelet reactivity was assessed using the VerifyNow^™^ P2Y12 assay.

**Results:**

Stent thrombosis occurred in 10 patients (3.52%), including 8 intraoperative and 2 delayed cases. KTT detected all intraoperative events, enabling immediate management such as conversion to balloon-assisted coiling. Among KTT-negative patients, delayed thrombosis occurred in 2 cases (0.7%). VerifyNow^™^ had no predictive value, as all thrombosis cases were classified as clopidogrel responders (≥50% inhibition). Multivariate analysis showed no association between thrombosis and sex, age, procedure duration, bleeding, clopidogrel timing, or VerifyNow^™^ inhibition. Thrombosis was, however, strongly linked to postoperative paresis or paralysis (*p* < 0.0001) and prolonged hospital stay (*p* = 0.0329).

**Conclusion:**

The Kazakh ThromboTest represents a practical and cost-effective intraoperative method with a high negative predictive value for identifying acute thrombotic risk during stent-assisted aneurysm repair. Despite adequate VerifyNow^™^ testing, KTT enabled timely procedural modifications, particularly in settings where standard platelet function assays are either unreliable or inaccessible. While VerifyNow^™^ did not predict thrombotic events, KTT contributed to safer procedural management and may serve as a valuable adjunct in neurointerventional practice.

## Introduction

The field of neurointerventional surgery, particularly the endovascular management of cerebral aneurysms, has evolved significantly over the past two and a half decades (1–3). Although simple coiling remains widely used, it carries higher recurrence rates than coiling with adjunctive devices such as stents or flow diverters. This difference is most pronounced in wide-neck or otherwise uncoilable aneurysms, where stent-assisted coiling or flow-diverter embolization offers a more effective treatment option (4). The incorporation of these devices has improved long-term occlusion rates but has concurrently introduced the risk of thromboembolic complications, necessitating the routine use of dual antiplatelet therapy (DAPT). Despite the widespread adoption of stent- and flow diverter-assisted techniques, antiplatelet protocols remain heterogeneous and lack standardized guidelines (5, 6).

The European Society of Cardiology (ESC) and the European Association for Cardio-Thoracic Surgery (EACTS) recommend a regimen of 75–100 mg/day of clopidogrel and 325 mg/day of aspirin for 5–7 days prior to the procedure (7). However, patient response to clopidogrel varies considerably, with resistance rates reported between 5 and 70% in some of the Asian communities (8–10).

This pharmacodynamic variability contributes to increased rates of stent thrombosis, reported in the literature to occur in 2.4 to 28% of cases (4).

To mitigate thromboembolic risk, several platelet function assays are employed preoperatively in specialized centers. Commonly used tests include the PFA-100, VerifyNow^™^ (Accriva Diagnostics, Inc.), and Multiplate Electrode Aggregometry (MEA). The VerifyNow^™^ assay measures platelet reactivity to antiplatelet therapy, defining inadequate P2Y12 inhibition as P2Y12 reaction units (PRU) < 240 (8) and insufficient aspirin effect as Aspirin Reaction Units (ARU) < 550.

Strategies to overcome resistance include administering additional clopidogrel loading doses, switching to an irreversible P2Y12 receptor antagonist such as prasugrel, or using a reversible, noncompetitive P2Y12 antagonist such as ticagrelor (11). Nevertheless, the clinical utility of these tests is limited by high costs, inconsistent availability, and questionable reproducibility.

In response to these limitations, the Kazakh ThromboTest (KTT) was developed as an intraoperative tool to assess thrombotic risk in real time by monitoring thrombosis formation within a partially deployed stent at 7- and 15-min intervals. By evaluating in-stent thrombus formation during the procedure, KTT offers a potentially valuable tool to optimize safety and guide immediate treatment adjustments in patients undergoing stent-assisted aneurysm repair. This study aimed to observe the development of intraoperative in-stent thrombosis, as an indicator of inadequate platelet inhibition, through the application of the Kazakh ThromboTest in a contemporary cohort of patients undergoing device-assisted treatment for cerebral aneurysms.

## Materials and methods

This retrospective study analyzed data from 284 consecutive patients who underwent stent-assisted endovascular treatment for cerebral aneurysms at a single neurovascular center in Kazakhstan between January 2020 and December 2023. All procedures were performed by experienced neurointerventional teams following standardized institutional protocols.

### Inclusion and exclusion criteria

Inclusion criteria comprised adult patients (≥18 years) undergoing elective or urgent stent-assisted coiling or flow diversion for intracranial aneurysms. Patients were excluded if they had incomplete clinical records, lacked VerifyNow platelet reactivity data, or experienced device failure unrelated to thrombosis.

### Antiplatelet therapy and testing

Preoperative coagulation profiles were routinely assessed, and VerifyNow™ P2Y12 testing was performed in all patients. Dual antiplatelet therapy (DAPT) consisted of aspirin (150 mg daily) and clopidogrel (75 mg daily) administered for 3–5 days before the procedure. If therapy was commenced only one day prior to surgery, a loading dose of clopidogrel (300–600 mg) was administered 24 h before intervention to ensure prompt platelet inhibition.

Platelet function was also evaluated intraoperatively using the VerifyNow™ P2Y12 assay (Werfen, United States), with both percentage inhibition and P2Y12 reaction units (PRU) values recorded. Clopidogrel resistance was defined as PRU > 240. Preprocedural VerifyNow™ confirmed adequate platelet inhibition in all patients or led to medication adjustment.

### KTT definitions

The Kazakh ThromboTest (KTT) is an intraoperative method for real-time assessment of antiplatelet therapy efficacy during stent-assisted procedures. The technique involves partial deployment of the stent (approximately 70–80% of its resheathing capacity) at the target site. Following this, serial check angiograms are performed at 7-min intervals over a 15-min observation period to evaluate for the development of in-stent thrombosis. A negative KTT—defined by the absence of thrombus formation—indicates adequate platelet inhibition, allowing the procedure to proceed with full stent deployment. A positive KTT, characterized by angiographic evidence of thrombus formation within the partially deployed stent, is considered a failure of antiplatelet therapy. In such cases, the stent is resheathed and removed, and an alternative treatment strategy is pursued.

### Definition of delayed thrombosis

Delayed thrombosis was defined as thrombotic vessel occlusion occurring more than 24 h after stent deployment. All cases of delayed thrombosis had a previously negative KTT result. Diagnosis was confirmed by follow-up digital subtraction angiography (DSA) or computed tomography angiography (CTA), or inferred from new-onset neurological deficits consistent with ischemia in the absence of technical failure or intraoperative thrombotic signs.

### Endovascular procedure

Following standard antiseptic preparation and induction of general anesthesia, vascular access was achieved via the common femoral artery utilizing a 7F introducer sheath. Neuroendovascular procedures involved the use of various braided stents (Leo/Leo Baby, Balt, France; LVIS/LVIS Jr., MicroVention, United States) and flow diverters (FRED, MicroVention, United States; Pipeline, Medtronic, United States; Silk, Balt, France; Derivo, Acandis, Germany). The Kazakh ThromboTest (KTT) was performed intraoperatively in 284 patients to assess real-time thrombotic risk.

If no thrombosis was observed, the stent deployment was completed, indicating presumed adequate platelet inhibition. A positive KTT—defined as visible in-stent thrombus—prompted stent removal, adjustment of antiplatelet therapy to an agent with a different mechanism of action (typically ticagrelor), and conversion to alternative strategies such as balloon-assisted coiling.

### Postoperative management and follow-up

Following the intervention, patients continued DAPT for 6 to 12 months, after which therapy was reduced to a single antiplatelet agent. Follow-up assessments included neurological evaluation at one month, MR angiography between 3–6 months, and digital subtraction angiography at 6 months. Additional imaging was conducted between 6 and 12 months as indicated by prior results.

### Statistical analysis

Data analysis included both descriptive and inferential statistics. Continuous variables, such as age, hospitalization days, and operation duration, were presented as medians with interquartile ranges and compared using the Wilcoxon two-sample test. Categorical variables, including sex, hypertension stage, stent type, aneurysm location and size, and post-procedural complications, were compared using Fisher’s exact test. Odds ratios (OR) with 95% confidence intervals (CI) were calculated to evaluate potential predictors of stent thrombosis. A *p*-value <0.05 was considered statistically significant. All statistical analyses were performed using SPSS version 26.0 (IBM Corp., Armonk, NY, United States), with full adherence to national and international ethical standards for data collection and reporting.

### Ethical considerations

The study received ethical approval from the institutional review boards of all participating centers. The approvals were granted as follows: Central Clinical Hospital JCI, Almaty, Kazakhstan. Approval Date: January 15, 2016. Protocol Number: 16.

## Results

Among 284 patients treated with stent-assisted endovascular therapy for cerebral aneurysms, stent thrombosis was observed in 10 cases (3.52%), including 8 intraoperative events detected by KTT and 2 delayed events. General demographic and clinical characteristics are summarized in Table 1. Patients originated from 19 different regions of Kazakhstan, with the majority residing in Almaty city (*n* = 137, 41.64%) and Almaty region (*n* = 66, 20.06%). There was no statistically significant difference in sex distribution between the groups (*p* = 1.0000). Hypertension was observed across all stages: AH1 in 40 patients (14%), AH2 in 25 patients (8.8%), and AH3 in 175 patients (61.6%). Normotension was present in 97.73% of non-thrombosis cases and 2.27% of thrombosis cases, with no statistically significant difference between groups (*p* = 0.4227).

**Table 1:** General characteristics.

	No thrombosis	Stent thrombosis	Total	*p*-value	Statistical test
Age	57.0 (48.0–63.0)	54.0 (46.0–58.0)	57.0 (48.0–63.0)	0,2,853	Wilcoxon Two-Sample Test
Sex					
Female	200 (96.15%)	8 (3.85%)	208	1	Fisher’s Exact test
Male	74 (97.37%)	2 (2.63%)	76		
Hypertension					
AH1	37 (92.5%)	3 (7.5%)	40	0,4,227	Fisher’s Exact test
AH2	24 (96%)	1 (4%)	25		
AH3	170 (97.14%)	5 (2.86%)	175		
No	43 (97.73%)	1 (2.27%)	44		
VerifyNow P2Y12 reaction units (PRU)					
Decreased platelet reactivity	263 (96.34%)	10 (3.66%)	273	1	Fisher’s Exact test
No drug persistent	11 (100%)	0 (0%)	11		
VerifyNow %					
< 50%	80 (97.56%)	2 (2.44%)	82	0,7,291	Fisher’s Exact test
50–100%	194 (96.04%)	8 (3.96%)	202		
Clopidogrel days before operation					
0	20 (90.91%)	2 (9.09%)	22	0,2,944	Fisher’s Exact test
1	230 (96.64%)	8 (3.36%)	238		
2	23 (100%)	0 (0%)	23		
3	1 (100%)	0 (0%)	1		
Paresis/paralysis post-op					
No	270 (99.63%)	1 (0.37%)	261	<0.0001	Fisher’s Exact test
Yes	4 (30.77%)	9 (69.23%)	23		

As shown in Table 1, there was no significant difference in median age between thrombosis and non-thrombosis groups (*p* = 0.2853). Female patients accounted for 80.0% of thrombosis cases and 96.15% of non-thrombosis cases, while males represented 2.63 and 97.37%, respectively. No significant differences were noted in hypertension distribution or other comorbidities.

Regarding the timing of antiplatelet administration, Clopidogrel was administered one day prior to the procedure in 8 of 10 thrombosis cases and in 96.64% of non-thrombosis cases, a difference that was not statistically significant (*p* = 0.2102). And on the day of operation the loading dose of Clopidogrel was given in to 22 patients, 2 of which developed stent thrombosis (9.09%).

To evaluate the predictive performance of intraoperative Kazakh ThromboTest (KTT) and preoperative VerifyNow^™^ P2Y12 Reaction Units (PRU) testing for intraoperative thrombosis, outcomes were analyzed in 284 patients. KTT was performed intraoperatively in all cases, yielding positive results in 8 patients (2.8%) and negative results in 276 patients (97.2%). Delayed thrombosis occurred exclusively in two KTT-negative patients (0.7%), as seen in Table 2. All patients underwent preoperative PRU testing. Using a 240-PRU threshold for clopidogrel resistance, 11 patients (3.9%) had PRU > 240, indicating resistance, and 273 patients (96.1%) had PRU ≤ 240. No thrombotic events occurred in the PRU > 240 group, corresponding to a positive predictive value (PPV) of 0% and a negative predictive value (NPV) of 100%. Among patients with PRU ≤ 240, two cases of delayed thrombosis were observed (0.7%), yielding an NPV of 99.25%, as shown in Table 2. All patients with positive intraoperative KTT test had stent removed and underwent balon-assisted coiling instead.

**Table 2:** Combined diagnostic matrix of KTT vs. VerifyNow^™^ PRU for predicting delayed thrombosis.

	KTT (+)	KTT (−)		Delayed Thrombosis (+)	Delayed Thrombosis (−)	
PRU > 240 (*n* = 11)	0	11	PPV 100%	0	11	PPV 0%
PRU ≤ 240 (*n* = 273)	8	265	NPV 99.25%	2	271	NPV 99.25%
Total	Sensitivity 80%	Specificity 100%		Sensitivity 0%	Specificity 96%	Total: 284

Cross-tabulation of KTT and PRU results demonstrated that all 10 thrombotic events occurred in patients with PRU ≤240. No thrombotic events were detected in patients with PRU >240, as presented in Table 2. *PRU: P2Y12 Reaction Units; KTT: Kazakh ThromboTest; PPV: Positive Predictive Value; NPV: Negative Predictive Value. A PRU threshold of 240 was used, with lower PRU values indicating better platelet inhibition.

Regarding the timing of antiplatelet administration, Clopidogrel was administered one day prior to the procedure in 5 of 10 thrombosis cases (50.0%) and in 257 of 274 non-thrombosis cases (93.8%), a difference that was not statistically significant (*p* = 0.2102). Eleven patients (3.9%) were identified as resistant and underwent regimen modification prior to treatment to ticagrelol with a loading dose of 180 mg, subsequently, they were continued at a dosage of 90 mg twice daily.

**Figure 1:**
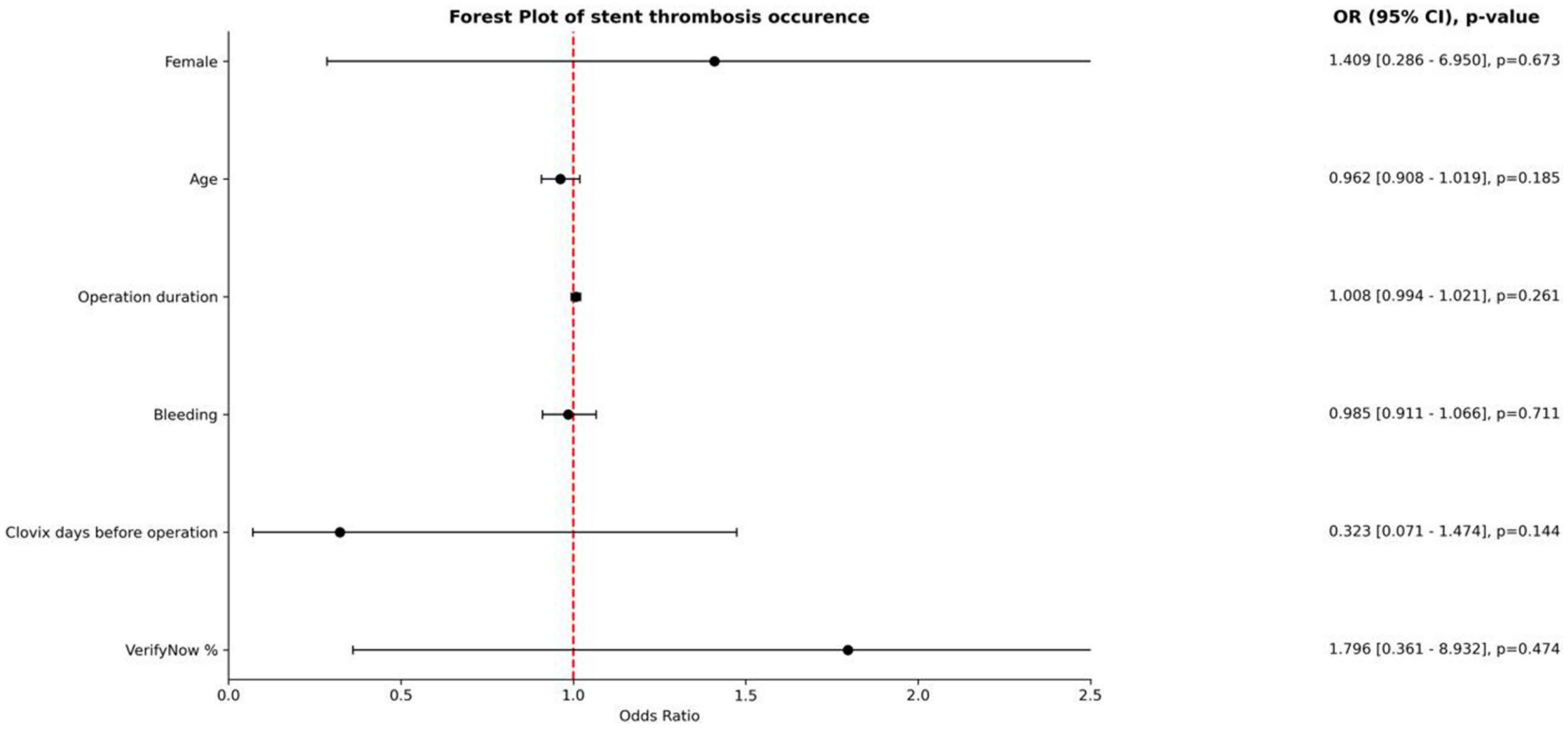
Forest plot illustrating odds ratios (ORs), 95% confidence intervals (CIs), and *p*-values for variables potentially associated with stent thrombosis. None of the examined parameters—sex, age, operation duration, bleeding volume, timing of Clopidogrel administration, or VerifyNow percentage inhibition—reached statistical significance. The red dashed line represents the null effect (OR = 1.0), and all confidence intervals intersect this line, indicating a lack of independent association with thrombosis occurrence.

A multivariable logistic regression analysis was performed to evaluate predictors of stent thrombosis (Figure 1). The forest plot shows that none of the examined clinical or procedural variables were statistically significant. Female sex (OR = 1.409; 95% CI: 0.286–6.950; *p* = 0.673), age (OR = 0.962; 95% CI: 0.908–1.019; *p* = 0.185), operation duration (OR = 1.008; 95% CI: 0.994–1.021; *p* = 0.261), and intraoperative bleeding (OR = 0.985; 95% CI: 0.911–1.066; *p* = 0.711) did not significantly affect thrombosis risk. Likewise, the number of days Clopidogrel was administered preoperatively (OR = 0.323; 95% CI: 0.071–1.474; *p* = 0.144) and VerifyNow percentage inhibition (OR = 1.796; 95% CI: 0.361–8.932; *p* = 0.474) were not predictive. All confidence intervals crossed the line of null effect (OR = 1.0), suggesting no single variable independently predicted thrombotic events in this cohort.

Table 3 presents a comparative analysis of procedural and anatomical variables in patients with and without stent thrombosis. Hospitalization duration was significantly longer in patients who developed thrombosis, with a median of 10 days (IQR 7–12) compared to 7 days (IQR 6–8) in non-thrombosis cases (*p* = 0.0329). However, no statistically significant differences were found in operation duration (*p* = 0.5673), recanalization success (*p* = 0.5926), or use of balloon assistance (*p* = 0.7026).

**Table 3:** Comparison of procedural and aneurysm characteristics between patients with and without stent thrombosis, which occured in delayed thrombosis group or in a group of +KTT patients.

	No thrombosis	Stent thrombosis	Total	*p*-value	Statistical test
Hospitalization days	7.0 (6.0–8.0)	10.0 (7.0–12.0)	7.0 (6.0–9.0)	0,0329	Wilcoxon Two-Sample Test
Operation duration (in minutes)	85.0 (65.0–120.0)	72.5 (65.0–110.0)	85.0 (65.0–120.0)	0,5,673	Wilcoxon Two-Sample Test
Recanalization					
No	251 (96.54%)	9 (3.46%)	260	0,5,926	Fisher’s Exact test
Yes	23 (95.83%)	1 (4.17%)	24		
Balloon assistance					
No	211 (96.79%)	7 (3.21%)	218	0,7,026	Fisher’s Exact test
Yes	63 (95.45%)	3 (4.55%)	66		
Artery					
ACA	7 (77.78%)	2 (20%)	9	0,0519	Fisher’s Exact test
Acom	21 (100%)	0 (0%)	21		
BA	11 (91.67%)	1 (8.33%)	12		
ICA	177 (97.79%)	4 (2.21%)	181		
MCA	57 (95%)	3 (5%)	60		
VA	1 (100%)	0 (0%)	1		
Stent type					
Derivo	19 (100%)	0 (0%)	19	0,7,645	Fisher’s Exact test
FRED	21 (95.45%)	1 (4.55%)	22		
LEO	130 (97.01%)	4 (2.99%)	134		
LVIS	49 (94.23%)	3 (5.77%)	52		
Pipeline	2 (100%)	0 (0%)	2		
SILK	53 (96.36%)	2 (3.64%)	55		
Aneurysm size					
Giant	20 (90.91%)	2 (9.09%)	22	0,2,388	Fisher’s Exact test
Medium	97 (97.98%)	2 (2.02%)	99		
Small	157 (96.32%)	6 (3.68%)	163		

As shown in Table 3, the anterior cerebral artery (ACA) was involved more frequently in patients with stent thrombosis compared to those without (20.0% vs. 2.21%), with this difference approaching statistical significance (*p* = 0.0519). The proportion of non-FD to FD stents was 65.4%. Among non-FD stents, LEO was the most frequently implanted device (*n* = 134), with 4 cases of thrombosis (2.99%), followed by LVIS (*n* = 52), which was associated with 3 thrombotic events (5.77%). In the FD group, different thrombosis rates were observed: SILK (*n* = 55) accounted for 2 cases (3.64%), while Derivo (*n* = 19), Pipeline (*n* = 2), and FRED (*n* = 22) were associated with either no events or only a single case of thrombosis. Although these differences did not reach statistical significance (*p* = 0.7645), LVIS and SILK devices demonstrated a relatively higher proportion of thrombotic events compared with the other stents.

### Hemorrhagic complications

Postoperative paresis occurred in 4 patients with hemorrhagic complications. These included: a 58-year-old male with a previously ruptured fusiform basilar artery aneurysm treated with LEO Baby stent–assisted coiling, complicated by arterial dissection and subarachnoid–intraventricular hemorrhage requiring ventriculostomy; a 56-year-old female with an ophthalmic segment ICA aneurysm treated with a SILK flow diverter; a 48-year-old female with massive SAH from an MCA bifurcation aneurysm treated with stent- and balloon-assisted coiling; and a 51-year-old female with SAH and vasospasm due to a giant choroidal segment ICA aneurysm treated with a Derivo flow diverter, nimotop, and balloon angioplasty.

## Case examples

See Figures 2, 3.

**Figure 2:**
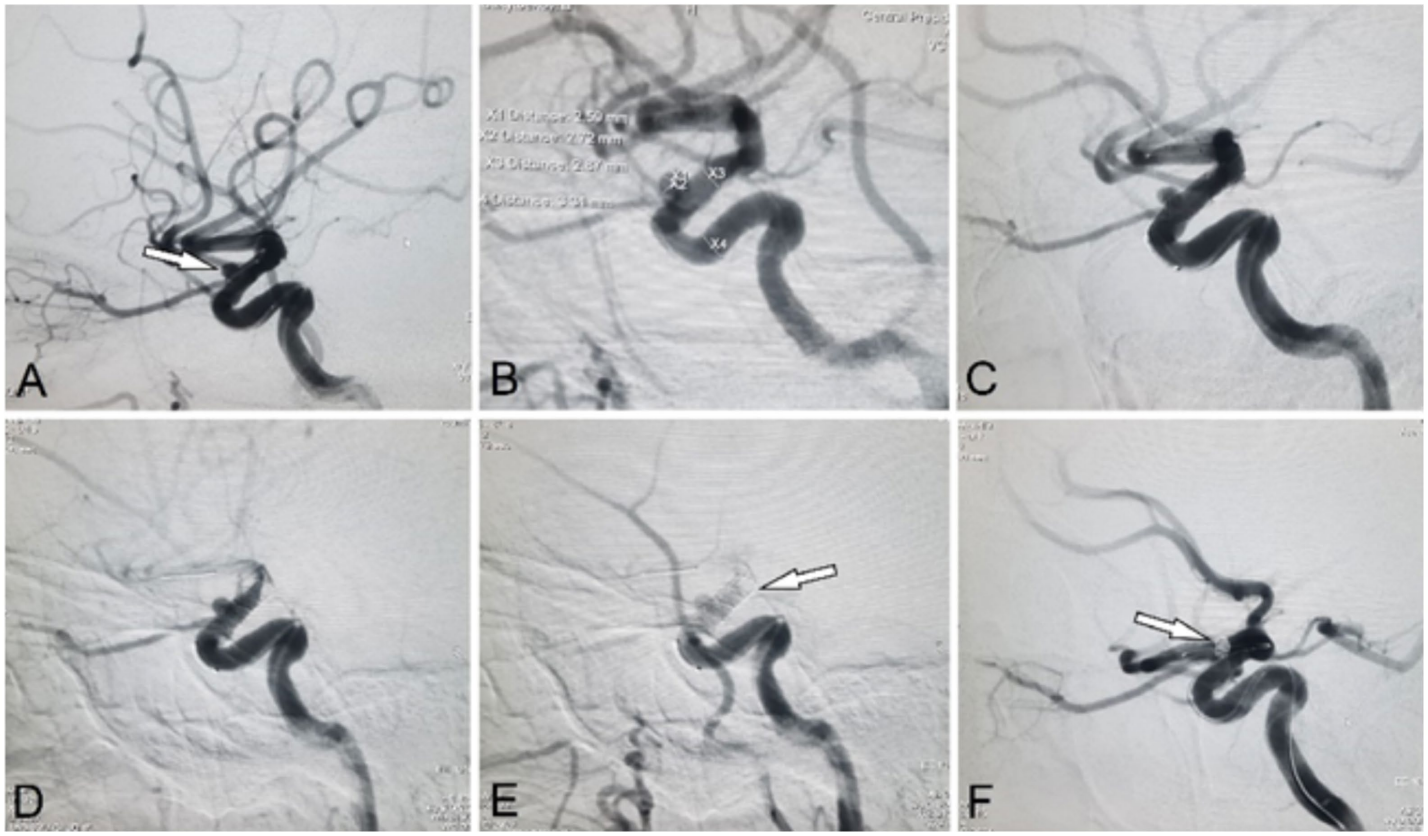
Representative case of a positive Kazakh ThromboTest (KTT) during an attempted internal carotid artery (ICA) stenting with a flow-diverter. **(A,B)** Angiographic views show a saccular aneurysm located at the C4 segment of the ICA. **(C)** Partial deployment of a self-expanding flow-diverter stent without full expansion. **(D)** Control angiography at 7 minutes post-deployment. **(E)** Follow-up at 14 minutes reveals a positive KTT with visible in-stent thrombosis and near-occlusion of the ICA. **(F)** Rescue coil embolization of the aneurysm following stent removal.

**Figure 3:**
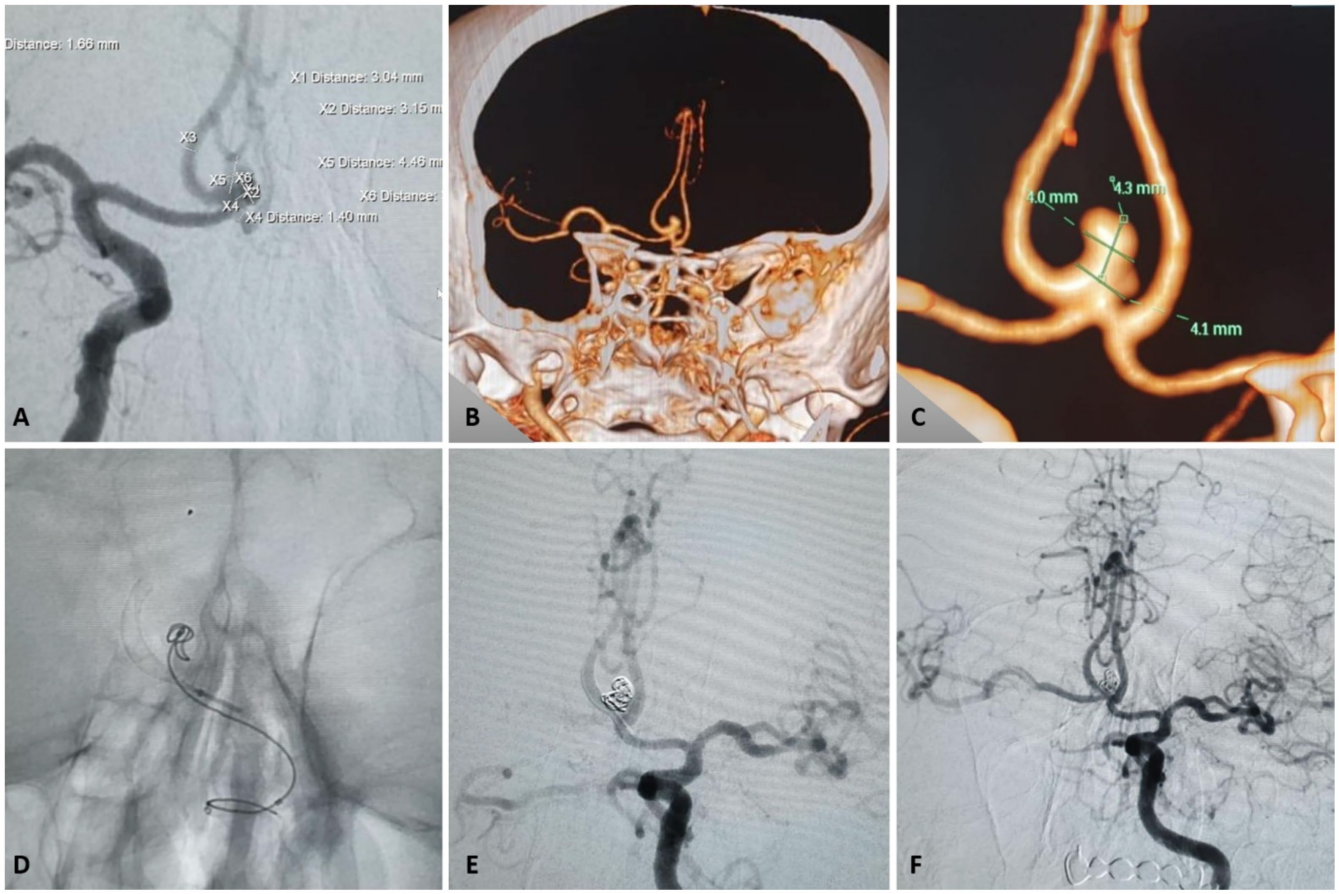
Clinical case illustrating a negative Kazakh ThromboTest (KTT) despite complete resistance on VerifyNow™ in the treatment of an anterior communicating artery (AComA) aneurysm using a P48 HPC flow diverter. **(A)** Frontal angiographic view showing a 4.4 × 4.0 × 3.1 cm AComA aneurysm. **(B,C)** Corresponding CT angiography images. **(D,E)** Intraoperative application of the KTT technique. **(F)** Successful deployment of the FRED device with no thrombotic complications.

## Discussion

The Kazakh ThromboTest (KTT) offers a practical intraoperative approach to mitigating thromboembolic complications during stent-assisted cerebral aneurysm treatment, particularly in settings where preoperative platelet function testing is either inaccessible or unreliable. Stent-assisted coiling is primarily employed for the management of giant or bifurcation aneurysms and may be performed using either flow-diverting (FD) or non-FD stents. In our series, the LEO stent was categorized as a non-FD device, although some reports have described it as exhibiting partial flow-diversion properties (12).

In this updated cohort of 284 patients, KTT identified hyperacute in-stent thrombosis in 8 out of 10 patients (80.0%), enabling immediate therapeutic adjustments such as stent removal or conversion to alternative techniques like balloon-assisted coiling. This real-time thrombotic risk assessment supports the growing role of individualized intraoperative antiplatelet management.

Notably, all 10 cases of stent thrombosis occurred despite VerifyNow^™^ results indicating adequate responsiveness to both aspirin and clopidogrel.

Although it is worth noting that regarding the timing of antiplatelet administration, Clopidogrel was administered one day prior to the procedure in 8 of 10 thrombosis cases and in 96.64% of non-thrombosis cases, a difference that was not statistically significant (*p* = 0.2102). And on the day of operation the loading dose of Clopidogrel was given in to 22 patients, 2 of which developed stent thrombosis (9.09%), and as studies on coronary stenting show, the loading dose of clopidogrel resulted in maximal antiplatelet efficacy 1 day after drug administration (13).

This underscores the limited predictive value of conventional preoperative platelet function testing in identifying patients at risk for acute or delayed thrombotic events. Such findings suggest that reliance on VerifyNow^™^ alone may provide a false sense of security, whereas intraoperative assessment with KTT can offer actionable, real-time data to guide immediate clinical decision-making.

Thromboembolic events remain a recognized complication of endovascular aneurysm treatment, often driven by suboptimal platelet inhibition due to P2Y12 receptor antagonist resistance (14). This challenge is particularly relevant in Central and Southeast Asia, where genetic variability and inconsistent access to platelet function assays contribute to higher resistance rates (15, 16). While dual antiplatelet therapy (DAPT) regimens are generally derived from cardiovascular protocols (6, 9), their translation to neurovascular practice is imperfect and frequently undermined by pharmacogenetic factors, poor compliance, and comorbidities (17–21).

Our findings reaffirm the value of KTT as an intraoperative screening tool: among the 284 patients tested, those with a negative KTT experienced no acute in-stent thrombosis during the procedure. However, 2 delayed thromboembolic events were recorded in KTT-negative cases (0.7%), emphasizing the temporal limitation of the test. These outcomes underscore that while KTT is highly informative for acute risk stratification, it cannot substitute for longitudinal surveillance or a comprehensive perioperative risk framework.

Conversely, VerifyNow^™^—despite its widespread use for preoperative platelet reactivity assessment—exhibited poor predictive utility in our cohort. None of the 10 patients who developed stent thrombosis had a VerifyNow inhibition value below the resistance threshold of <50%, resulting in a sensitivity and positive predictive value of 0%. The assay’s specificity was modest (70.75%), and its negative predictive value was high but not definitive. These findings call into question the clinical assumption that a higher VerifyNow percentage (≥50%) equates to lower thrombotic risk, especially in neurointerventional contexts.

This discrepancy illustrates the complementarity—but not equivalence—of biochemical and procedural assessments, as KTT may provide a more direct reflection of the real-time hemodynamic and thrombotic environment than static preoperative assays. In case of a false-positive KTT, the stent was fully deployed before 15 min, and the patient’s robust collateral circulation provided adequate safety. The absence of visible thrombus during intraoperative angiography in KTT-negative patients further supports the test’s reliability. Nonetheless, false negatives remain a concern. The protocol’s reliance on 7- and 15-min observation intervals following partial stent deployment may fail to capture slowly developing thrombi or subtle microembolic signals, particularly in patients with strong collateral flow or delayed platelet activation (22–26). These technical limitations must be acknowledged when interpreting a negative KTT result.

Moreover, the partial stent deployment inherent to the KTT protocol may itself induce local flow disturbances and intimal stress, potentially leading to thrombus formation even in patients with adequate platelet inhibition (27). The study by Rouchaud et al. demonstrated that good wall apposition was a key factor for aneurysm occlusion after flow diverter treatment in a histological evaluation of rabbits (28). This effect could overestimate the thrombotic predisposition of some patients and should be considered when interpreting positive KTT results. The structured KTT protocol is designed to identify such risk intraoperatively; however, continued clinical vigilance during the postoperative period remains crucial. Considering the high morbidity associated with thromboembolic complications, integration of KTT into procedural workflows may enhance patient safety, particularly in scenarios necessitating rapid transition from clopidogrel to alternative agents such as ticagrelor or prasugrel (21, 22). It is also important to consider that elevated blood pressure during testing could potentially influence thrombotic dynamics and warrants further investigation (26).

The current analysis supports deferring stent deployment in KTT-positive cases and opting for safer alternatives such as balloon-assisted coiling, simple coiling, or microsurgical clipping. Alternatively, intraoperative modification of the antiplatelet regimen, including switching to more potent agents, may be considered. This adaptive strategy is particularly critical in patients with uncertain antiplatelet responsiveness or anatomical features that increase the risk of thrombosis.

These findings echo previous literature demonstrating improved durability of occlusion with stent-assisted techniques (15, 16), while stressing the need for optimized antiplatelet management to prevent ischemic complications. Although extended DAPT enhances protection against thrombosis, it also raises the risk of hemorrhagic events. Hence, an individualized transition to single antiplatelet therapy, based on aneurysm healing and endothelialization, is vital (21).

Despite its strengths, this study has notable limitations. Its retrospective design introduces potential selection and information bias. The KTT protocol inherently prevents direct evidence of whether a stent with a positive test result would subsequently thrombose if fully released or replaced by another device, since all such stents were resheathed and withdrawn. Consequently, our findings rely on indirect inference rather than on documented *in vivo* outcomes of implanted KTT-positive stents. The variability in stent types, operator technique, and antiplatelet protocols may confound generalizability. Importantly, not all patients had complete preoperative VerifyNow data, limiting the comparison between biochemical and procedural testing.

A subset of patients received only a 300 mg clopidogrel loading dose 24 h before the procedure, rather than the standard 5-day regimen of 75 mg daily, which may have influenced platelet inhibition levels and affected the results. Additionally, KTT was not compared with other platelet function assays, such as Multiplate or platelet function analyser-100 (PFA-100), which limits the scope of the validation.

Future research should focus on prospective, multicenter trials comparing KTT with validated biochemical tests, along with implementation of high-resolution imaging modalities to detect microthrombosis. Parallel exploration of stent design and biocompatibility innovations will further improve outcomes. In resource-limited settings, the KTT offers a cost-effective, reproducible, and clinically actionable tool to improve patient safety during complex neuroendovascular procedures.

## Conclusion

The Kazakh ThromboTest (KTT) demonstrated clinical utility as a simple and effective intraoperative tool for identifying thrombotic risk during stent-assisted treatment of cerebral aneurysms. In this updated cohort, KTT enabled real-time detection of hyperacute in-stent thrombosis in 80% of affected patients, facilitating timely adjustments such as stent removal or conversion to alternative strategies. Overall clinical thrombosis was 7.4% in this series.

Notably, no intraoperative thrombotic events occurred among KTT-negative patients, reinforcing the test’s reliability in ruling out immediate risk.

However, the occurrence of delayed thromboembolic complications in a small subset of KTT-negative patients underscores the temporal limitations of the test and the need for continued postoperative vigilance. Given its high negative predictive value, low cost, and ease of implementation, KTT represents a valuable adjunct to standard procedural protocols, particularly in settings with limited access to advanced platelet function testing.

In cases where KTT indicates thrombotic risk, stent deployment should be deferred in favor of safer alternatives such as balloon-assisted coiling, simple coiling, or microsurgical clipping. Future studies should aim to refine KTT timing and interpretation, investigate its role alongside biochemical assays, and explore integration with high-resolution imaging to further optimize neurointerventional safety and outcomes.

## Data Availability

The raw data supporting the conclusions of this article will be made available by the authors, without undue reservation.
